# Co-Localized or Randomly Distributed? Pair Cross Correlation of *In Vivo* Grown Subgingival Biofilm Bacteria Quantified by Digital Image Analysis

**DOI:** 10.1371/journal.pone.0037583

**Published:** 2012-05-24

**Authors:** Claudia Schillinger, Annett Petrich, Renate Lux, Birgit Riep, Judith Kikhney, Anton Friedmann, Lawrence E. Wolinsky, Ulf B. Göbel, Holger Daims, Annette Moter

**Affiliations:** 1 Institut für Mikrobiologie und Hygiene, Charité – Universitätsmedizin Berlin, Berlin, Germany; 2 UCLA School of Dentistry, University of California Los Angeles, Los Angeles, California, United States of America; 3 Abteilung für Parodontologie und Synoptische Zahnmedizin, Charité – Universitätsmedizin Berlin, Berlin, Germany; 4 School of Dentistry, Faculty of Health, University of Witten, Witten, Germany; 5 Texas A&M Health Science Center, Baylor College of Dentistry, Dallas, Texas, United States of America; 6 Department of Microbial Ecology, Ecology Center, University of Vienna, Vienna, Austria; University of Liverpool, United Kingdom

## Abstract

The polymicrobial nature of periodontal diseases is reflected by the diversity of phylotypes detected in subgingival plaque and the finding that consortia of suspected pathogens rather than single species are associated with disease development. A number of these microorganisms have been demonstrated *in vitro* to interact and enhance biofilm integration, survival or even pathogenic features. To examine the *in vivo* relevance of these proposed interactions, we extended the spatial arrangement analysis tool of the software *daime* (*digital image analysis in microbial ecology*). This modification enabled the quantitative analysis of microbial co-localization in images of subgingival biofilm species, where the biomass was confined to fractions of the whole-image area, a situation common for medical samples. Selected representatives of the disease-associated red and orange complexes that were previously suggested to interact with each other *in vitro* (*Tannerella forsythia* with *Fusobacterium nucleatum* and *Porphyromonas gingivalis* with *Prevotella intermedia*) were chosen for analysis and labeled with specific fluorescent probes via fluorescence *in situ* hybridization. Pair cross-correlation analysis of *in vivo* grown biofilms revealed tight clustering of *F. nucleatum/periodonticum* and *T. forsythia* at short distances (up to 6 µm) with a pronounced peak at 1.5 µm. While these results confirmed previous *in vitro* observations for *F. nucleatum* and *T. forsythia*, random spatial distribution was detected between *P. gingivalis* and *P. intermedia* in the *in vivo* samples. In conclusion, we successfully employed spatial arrangement analysis on the single cell level in clinically relevant medical samples and demonstrated the utility of this approach for the *in vivo* validation of *in vitro* observations by analyzing statistically relevant numbers of different patients. More importantly, the culture-independent nature of this approach enables similar quantitative analyses for “as-yet-uncultured” phylotypes which cannot be characterized *in vitro*.

## Introduction

Periodontal diseases are prevalent bacterial biofilm infections in humans that involve progressive destruction of the tooth-supporting tissues and ultimately tooth loss in the absence of treatment. A strong association between periodontal and systemic diseases as well as unfavorable pregnancy outcomes has also been reported [1], [2], [3]. The traceability of periodontal bacteria and derived concepts of pathogenesis, however, relate strongly to the methods applied for microbial analysis.

Early electron microscopy studies revealed the complexity and highly organized structure of the microbiota residing in subgingival biofilms [4], [5], [6], [7]. Further efforts to elucidate the etiology of periodontal diseases included: **(i)** A comprehensive inventory of the oral microbiome: To date more than 1,000 distinct taxa have been identified in the oral cavity and about 400 of these have been so far associated with the colonization of the periodontal pocket [8], [9]. In addition to species identification, a number of these culture-independent studies implicated novel periodontal pathogens [10], [11], [12], [13], [14], [15]; **(ii)** Arrangement of a large panel of cultivable subgingival flora into microbial complexes based on their co-occurrence and association with health and disease [16]: Reflective of the multitude of microorganisms comprising the oral microbiota, clusters of microorganisms rather than single species have been implicated as indicators for periodontal health or disease. The so-called “red complex” which is strongly correlated with the severity of disease is comprised of *Porphyromonas gingivalis*, *Tannerella forsythia*, and *Treponema denticola*. These species appear to require the more prevalent “orange complex” species such as *Fusobacterium spp.* or *Prevotella intermedia* among others for biofilm integration [17]; **(iii)** Extensive *in vitro* examination of the ability of oral species to form aggregates with each other [18], [19], [20], [21], [22], [23], [24], [25], [26], [27]: The multitude of *in vitro* studies assessing individual interspecies adherence behavior or “co-aggregation” allowed a more detailed picture of the elaborate interactions involved in building the architecturally complex oral biofilm networks. Some of these *in vitro* interactions were validated *in vivo* for supragingival biofilm formation [28], [29], [30]. The subgingival *in vivo* distribution of several residents of the periodontal pocket has been examined immuno-histochemically [31], [32] and most recently via a very comprehensive fluorescent *in situ* hybridization (FISH)-based study [33]. Taken together these approaches cumulated in our current understanding that periodontal diseases involve complex synergistic and antagonistic bacterial interactions [34].

In contrast to diseases caused by a single etiological agent, polymicrobial biofilm infections are characterized by multiple, often opportunistic pathogens whose virulence features are often enhanced by the interplay with other community members. Therefore, the subgingival *in vivo* dynamics and bacterial biofilm interactions have become a focus of current periodontal research. Three critical issues remain to be addressed: **(i)** the casting of main characters is currently incomplete: even though certain oral microbial species have been assigned as periodontal colonizers the disease-association of the majority of species still has to be revealed [10], [12], [13], [35], [36]; **(ii)** The *in vivo* pathogenic potential of specific bacteria (“casting of good and bad guys”) continues to be under discussion, especially since the complexity of the biofilm network enables mutual interactions that we are just beginning to comprehend [10], [14], [37]; **(iii)** Finally, the “leading and supporting players” in the interplay of this lively, interwoven network of subgingival plaque bacteria are hardly determined [38]. Especially co-localization can be indicative of cell-to-cell adherence or synergistic associations. Detailed understanding of these complex relationships is adamant for the development of comprehensive therapeutical concepts targeting key pathogenic species or interactions, one of the central goals to improve the existing therapies [10], [15], [36], [39]. The majority of current studies, however, are limited to a qualitative assessment of species distribution which can be very subjective and quantitative confirmation of bacterial interactions are still lacking. Recent work by Valm and coworkers [40] presents an important step in this direction by examining the proportion of certain bacterial species that directly touch each other in dispersed dental plaque with combinatorial labeling and spectral imaging fluorescence *in situ* hybridization (FISH).

Our present study provides novel insight into the spatial relationships among bacteria including adhesion-based events as well as those based on metabolic relationships in a naturally grown, subgingival biofilm on a quantitative level. A recently established carrier-based *in vivo* model [41] enabled sampling of undisturbed subgingival biofilm and hybridized sections allowed high resolution examination on a single cell level. Two pairs of suspected periodontal pathogens were visualized by fluorescence *in situ* hybridization (FISH) and their distribution relative to each other was digitally quantified. For this proof of concept study, target species were chosen on the basis of the associations defined by Socransky's microbial complexes in subgingival biofilms [17] and positive interactions determined *in vitro* [19]. Since *T. forsythia*, a member of the strongly periodontitis-associated red complex, and *F. nucleatum* of the orange complex physically and synergistically interrelate *in vitro* [26], we examined the *in vivo* relevance of their relationship on a quantitative level. Additionally, the controversial relationship between the suspected periodontal pathogens *P. gingivalis* (red complex) and *P. intermedia* (orange complex) was evaluated. These oral bacterial species have been found to adhere to each other by some authors [42], while others imply that they do not interact [23], [43].

In the present study the target organism pairs were first visualized by FISH and epifluorescence microscopy for identification and analysis of their localization within the histological context. Second, the pair cross-correlation function (PCC) was quantified to determine whether the pairwise spatial arrangement of the analyzed bacterial populations was random, attractive or repulsive. For this purpose, we extended the spatial arrangement analysis tool of the software *daime*, “Digital Image Analysis In Microbial Ecology” [44]. For the quantification of spatial arrangement patterns, *daime* implements a stereological approach to estimate the PCC [45]. The generated PCC curve allows the determination of co-localization, random distribution or rejection (mutual avoidance) of two bacterial populations. This concept has successfully been applied to environmental biofilms [44], [46], [47] and to *in vitro* grown dental biofilm bacteria [48] and now for the first time could be applied to validate relationships of oral bacterial species in medical biofilms *in situ*.

## Methods

### Ethics Statement

The study was approved by the local Institutional Review board, the Ethikkommission der Charité-Universitätsmedizin Berlin and written consent of the participants was obtained.

### Subject population

Ten previously untreated subjects (three male and seven female) with generalized aggressive periodontitis (GAP) selected from a population referred for periodontal treatment to the Department of Periodontology at the University Hospital Charité were included in this institutionally approved study. Subjects ranged in age between 18 and 44 years (mean 35.1, SD 7.3 years). Clinical examination included medical and dental history, intraoral examination, full-mouth periodontal probing as well as a full mouth series of intraoral radiographs. Inclusion criteria for patient selection were based on the diagnosis of GAP according to the criteria of the 1999 International Workshop for Classification of Periodontal Disease and Conditions [49]: disease onset estimated at <30 years based on clinical examination, past radiographs, and/or interview, as well as 6 mm probing pocket depth (PPD) at a minimum of three permanent teeth other than first molars and incisors. Exclusion criteria were previous periodontal treatment, chronic systemic disease, anti-inflammatory or antimicrobial therapy within the last six months as well as pregnant or lactating women.

### Sampling

Subgingival biofilms were grown *in vivo* using a carrier-based model system as described previously [41]. Briefly, carriers were inserted in periodontal pockets of 10 GAP patients in 28 sample sites with a mean periodontal probing depth (PPD) of 7.8 mm, SD 1.3 mm. After 7 days of biofilm development, carriers were fixed, embedded, and sectioned as described previously [50]. Sections (2 µm in thickness) were sliced along the longitudinal axis of the e-PFTE carrier [41].

### Fluorescence *in situ* hybridization (FISH)

Probes for detection of *F. nucleatum/periodonticum* (FUNU), *T. forsythia* (TAFO, formerly named B(T)AFO)), *P. gingivalis* (POGI) as well as *P. intermedia* (PRIN), and the domain-specific probe EUB338 which recognizes most *Bacteria* were synthesized commercially (Biomers, Ulm, Germany). These probes have been published previously and were deposited in probeBase [51]. The sequence of probe FUNU matches those of *F. nucleatum*, *F. periodonticum*, *F. naviforme*, and *F. canfelinum*, the latter two not being relevant for periodontal disease. The species-specific probes were 5′end-labeled with either the Cy3 (indocarbocyanine) or Cy5 (indodicarbocyanine) fluorescent dye, while EUB338 contained FITC (Fluoresceinisothiocyanate) as a label to allow combinations with each species-specific probe. FISH procedures were performed as reported previously [52]. To confirm the specifity of the probes, fixed cells of the following strains served as positive controls: *F. nucleatum* (ATCC 25586), *P. gingivalis* (ATCC 33277), *P. intermedia* (ATCC 25611) and *T. forsythia* (ATCC 43037); species with the lowest number of mismatches at the probe binding site served as negative control: respectively *F. varium* (ATCC 8501), *P. gulae* (ATCC 51700), *P. bryantii* (DSM 11371), *B. suis* (ATCC 35419). Vectashield (Vector Laboratories, Orton Southgate, UK) was applied as mounting medium containing DAPI (4′,6-Diamidino-2-phenylindole) for visualization of all cells including eukaryotic cell nuclei.

### Epifluorescence microscopy and image acquisition

Microscopic observations were performed with an epifluorescence microscope (AxioPlan II, Zeiss, Jena, Germany). The microscope was equipped with a 100 W high-pressure mercury lamp (HB0 103W/2; Osram, Munich, Germany) and 10×, 40× and 100× objectives. Narrow band filter sets (AHF Analysentechnik, Tübingen, Germany) were applied to separate the FITC (ET F46-002) -, Cy3 (HQ F41-007) -, Cy5 (HQ-F41-008)-and DAPI (HQ F31-000)-derived signals, respectively. For image acquisition, the AxioPlan microscope was combined with an AxioCam MRm (Zeiss) digital camera controlled by the AxioVision 4.7 software. Multichannel images were captured at 1000× magnification and a resolution of 1388×1040 pixels (16 bit). For each patient and bacterial pair-combination the entire hybridized sections were examined and images were recorded at random positions, for fields of view (FOV) in which both species of interest were present. The location of each single image was mapped in an overview of the entire section.

### Image processing and analysis

**Figure 1:**
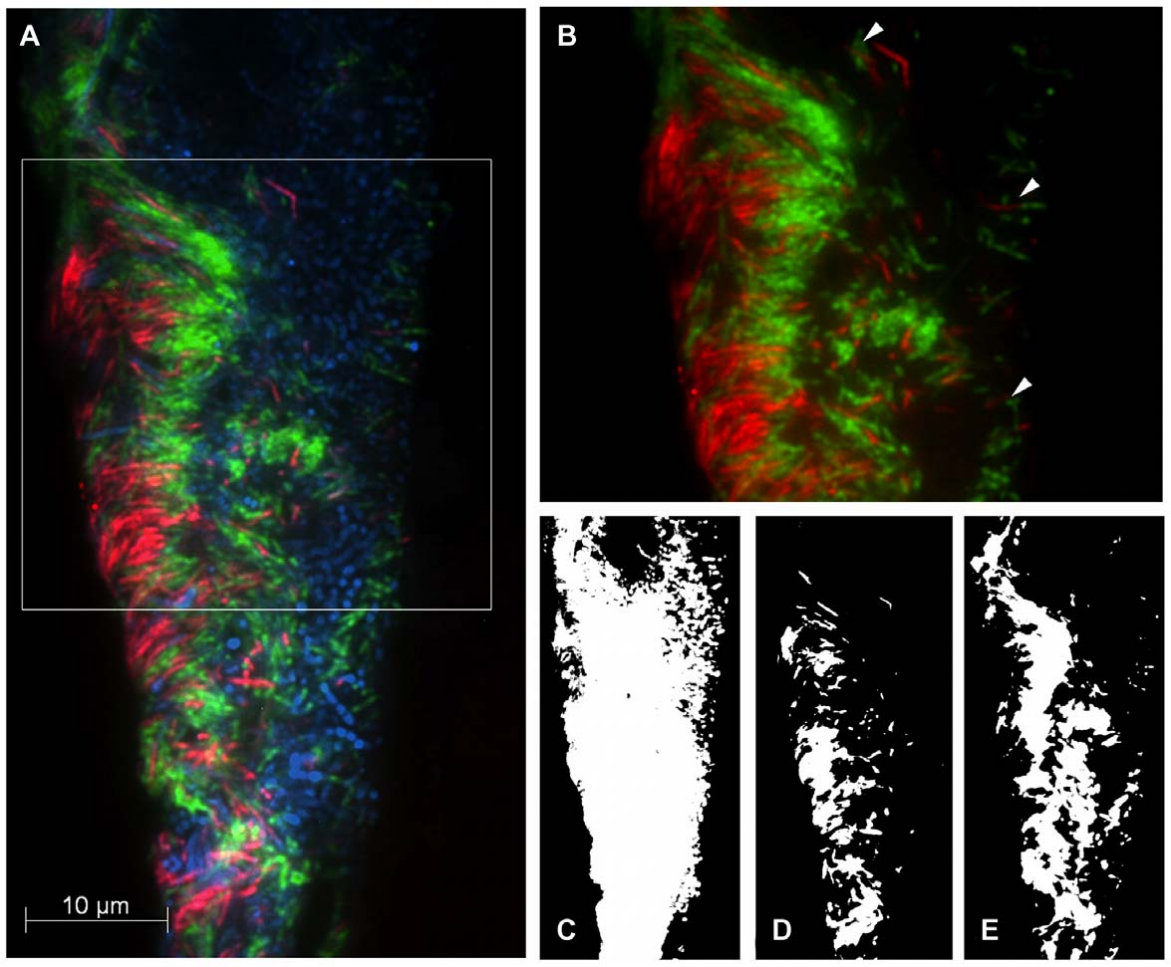
Representative micrograph of spatial interaction between *T. forsythia* and *F. nucleatum/periodonticum*. FISH was performed on sections of 7-day-old subgingival biofilm grown on e-PTFE carriers in gingival pockets of GAP patient 01. *T. forsythia and F. nucleatum/periodonticum* were fluorescently labeled with the species-specific probes TAFO-Cy3 (red) and FUNU-Cy5 (green), respectively. The spatial expansion of the entire biofilm was revealed with the domain-specific probe EUB338-FITC (blue). (A) The overlay of the Cy3, Cy5 and FITC channels shows the periodontal plaque between the gingival surface at the right edge and the carrier surface on the left. *F. nucleatum/periodonticum* (green) is densely packed together with *T. forsythia* (red) and appear to co-localize within an amorphous interwoven cluster focused on the carrier adjacent side. (B) Species-specific channels Cy3 and Cy5 in higher magnification. Single cells of *T. forsythia*, dispersed in the middle and right part of the biofilm, were found in close contact to *F. nucleatum/periodonticum* cells (arrowheads). (C–E) shows the respective binary masks of the micrograph (A) prepared for *spatial arrangement analysis* provided by the software *daime*. (C) The EUB338-FITC-channel served as reference mask to limit the calculation to the area of the biomass. (D–E) Between the segmented masks of species-specific channels TAFO-Cy3 (D) and FUNU-Cy5 (E) *daime* calculated the pair cross correlation function *g(r)*.

**Figure 2:**
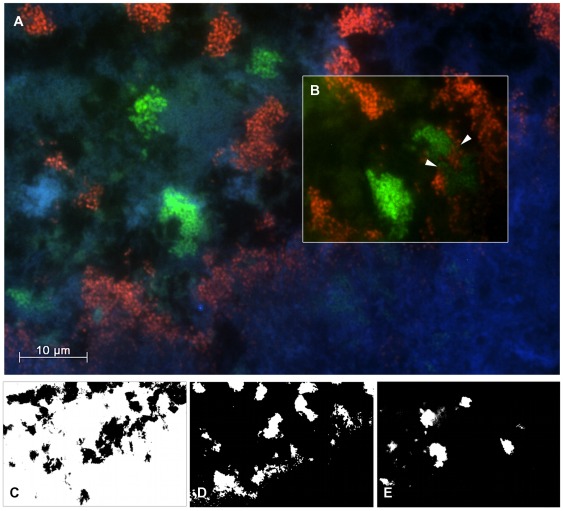
Visualization of the spatial arrangement of *P. gingivalis* and *P. intermedia*. FISH analysis performed on a carrier of GAP patient 05 reveals *P. gingivalis* in combination with *P. intermedia* respectively detected by the probes POGI-Cy5 (green) and PRIN-Cy3 (red). The domain-specific probe EUB338-FITC (blue) displays the entire biofilm expanded between the gingival surface at the bottom of the image and the carrier surface at the top. (A) An overlay of Cy3, Cy5 and FITC channels shows discrete microcolonies of *P. gingivalis* and *P. intermedia* apparently equispaced except the part marked by arrowheads. (B) In a punctual area species-specific channels Cy3 and Cy5 reveal both bacteria closely intermingled. (C–E) shows the respective binary masks of the micrograph (A) prepared for statistical quantification of the spatial relationship. (C) The EUB338-FITC-channel served as reference mask to limit the calculation to the area of the biomass. (D–E) Between the segmented masks of species-specific channels PRIN-Cy3 (red) and (E) POGI-Cy5 (green) *daime* calculated the pair cross correlation function *g(r)*.

Export: The images were exported in the grayscale uncompressed tagged image file format (TIFF). At least 25 independent images were combined to separate sets for FITC, Cy3 and Cy5, respectively, for each patient and pair of probes (Figures 1 C–E and Figures 2 C–E). These image sets were further processed by the procedures detailed below.

Image binarization: The segmentation of the 2D-images into objects (cells or clusters of cells) required the conversion of grayscale pictures into binarized images by luminance thresholding. In the binarized images, white pixels represented biomass, whereas black pixels were background. Strong variations in brightness and contrast between the different micrographs comprising an image set required the use of individual luminance thresholds for the univocal classification of biomass and background in each image. For this purpose, the effect luminance key of the program After Effects 5.5 (Adobe Systems) was employed to set a manual background threshold in each micrograph. Binarized images were saved in the TIFF format.

Spatial arrangement analysis with *daime*: *daime* is an Open Source software for digital image analysis of microbial cells in situ. The three sets of binarized TIFF-images obtained per patient and per pair of bacterial populations, which corresponded to the total biomass (FITC-labeled) and the two microbial species of interest (Cy3 and Cy5-labeled), were imported into *daime* for further analysis. The xy-µm size of the images was set to 88.31×66.92 µm according to the scaling factor of 0.064 µm/pixel indicated by the AxioVision Software. Automatic 2D-segmentation was performed to identify connected components (i.e., objects such as microbial cells and cell aggregates). During this step objects smaller than 28 pixels, which most likely represented noise, were ignored. Remaining artifacts, human or abiotic materials were rejected using the object editor options. To perform the spatial arrangement analysis by the “Linar Dipole” algorithm, the distance range was set from 0 to 50 µm and every fifth distance spaced at intervals of ∼0.5 µm was selected. The analysis was performed in random dipole mode, where the number of random dipoles was adjusted to 200,000 per distance. Finally, the reference space for the analysis was specified, individually for each FOV, by using the EUB338 images as reference space masks. The results were imported into Microsoft Excel (Microsoft Corporation).

The spatial arrangement tool provided with the software *daime* applies a stereological method to estimate the pair correlation (for one microbial population) or the pair cross-correlation (for two populations) functions by analyzing the chance encounters between cells and linear dipole probes. This approach has been described in detail elsewhere (Daims et al. 2006, [53]). The obtained PCC, g(r), indicates whether two populations co-aggregate, avoid each other, or are randomly distributed at distance r (in µm). Random distribution (the ‘null hypothesis’) is indicated by g(r) = 1, whereas g(r)>1 suggests co-aggregation and g(r)<1 mutual avoidance of the populations.

### Statistical analysis

Statistical validation of spatial arrangement analysis was executed by *daime* with n≥25 images per pair of bacteria and per patient. For n images the mean PCC g(r) for each distance r and 95% confidence interval (CI) was calculated using the standard deviation among the images and the student's t-distribution for n-1 degrees of freedom [44]. The data obtained for all patients were then merged individually for TAFO/FUNU and POGI/PRIN, respectively, by statistical evaluations to enable comparison of the results. This consolidation of the PCC results was performed by calculating a common PCC curve for each bacterial pair. Since the data were normally distributed, group mean (m) and standard error of the mean (SEM) were used to calculate CI for the group mean by the formula 95% CI = m ±1.96* SEM.

## Results

### Extension of the spatial arrangement tool of *daime*

The *in vivo* grown subgingival biofilms analyzed in this study were restricted in size and shape by a number of factors (population densities, spatial limits such as the intrinsic margins defined by the periodontal pocket and carrier etc.). Thus, the images of the biofilm sections contained biomass plus variable proportions of empty space (background), the latter mainly beyond the natural borders of the biofilm. The software *daime* [44], which we employed for quantifying the spatial localization of bacterial populations, was previously used in studies of biofilms and activated sludge flocs from wastewater treatment plants [44], [46]. The abundant biomass in those samples typically filled the entire FOV, and the recorded FISH images did not contain much (if any) empty space. Hence, the original version of the spatial arrangement analysis tool in *daime* used the whole images as “reference space” for the Linear Dipole algorithm [44]. This approach would cause biases with the images of subgingival biofilm, because the spatial clustering of all biomass (enforced by the biofilm size and shape) in the non-empty regions of these images would result in high PCC values [*g*(*r*)>1] and could not be distinguished from biologically caused co-localization. To overcome this problem, an additional feature was added to *daime* that allows the user to define those image regions, which actually contain any biomass and should be used as the “reference space” in the analysis. For this purpose, the user can specify a so-called “reference space mask” image that indicates the locations of biomass and background. In this study, the images of the EUB338 FISH signal were used as such masks, because these images showed fluorescent signals in all biomass-containing regions. With this extension, the user can choose whether the whole images or only the regions defined by the mask must be considered as “reference space”. Tests with artificial images and with images of real biofilms from different sources confirmed that the Linear Dipole algorithm, when combined with mask images, yielded correct results even if biomass covered the images only partially (data not shown; for a review refer to Daims and Wagner [53]). The extended algorithm has been included in *daime* since version 1.2, which was used to produce the data sets presented in the following section.

### Spatial arrangement analysis of target species

The bacterial pairs *T. forsythia* with *F. nucleatum/periodonticum* and *P. gingivalis* with *P. intermedia* were chosen for this study. Each pair consists of a representative of the “red complex” (*T. forsythia* and *P. gingivalis*) and the “orange complex” (*F. nucleatum* and *P. intermedia*). While positive interactions between *T. forsythia* and *F. nucleatum* have been consistently reported [26], [33], [54], [55] but has never been confirmed by quantitative spatial analysis using *in vivo*-grown subgingival biofilms, the relationship between *P. gingivalis* and *P. intermedia* is less clear [23], [42], [43]. Their spatial *in vivo* association was examined via qualitative visual inspection followed by quantitative spatial arrangement analysis to determine, by means of the PCC function, the co-localization, repulsion or randomness of bacterial distribution.

### Analysis of *T. forsythia* and *F. nucleatum/periodonticum*

Among the 10 subjects, 22 subgingival plaque carriers obtained from eight different patients exhibited strong hybridization signals for the probe combination TAFO/FUNU (detection of *T. forsythia*/*F. nucleatum/periodonticum*). FISH results of the remaining patients were either negative for the respective probes, or the fluorescent signals exhibited a low signal to noise ratio which made them unsuitable for further analysis. Visual inspection revealed excellent single cell resolution with typical morphologies of the target species among a variety of different bacterial morphotypes. A typical pattern of colony and cell association was observed in numerous specimens hybridized with TAFO/FUNU; strongly suggesting co-localization of these species (Figure 1).

### Individual-related spatial analysis of *T. forsythia* and *F. nucleatum/periodonticum*

**Figure 3:**
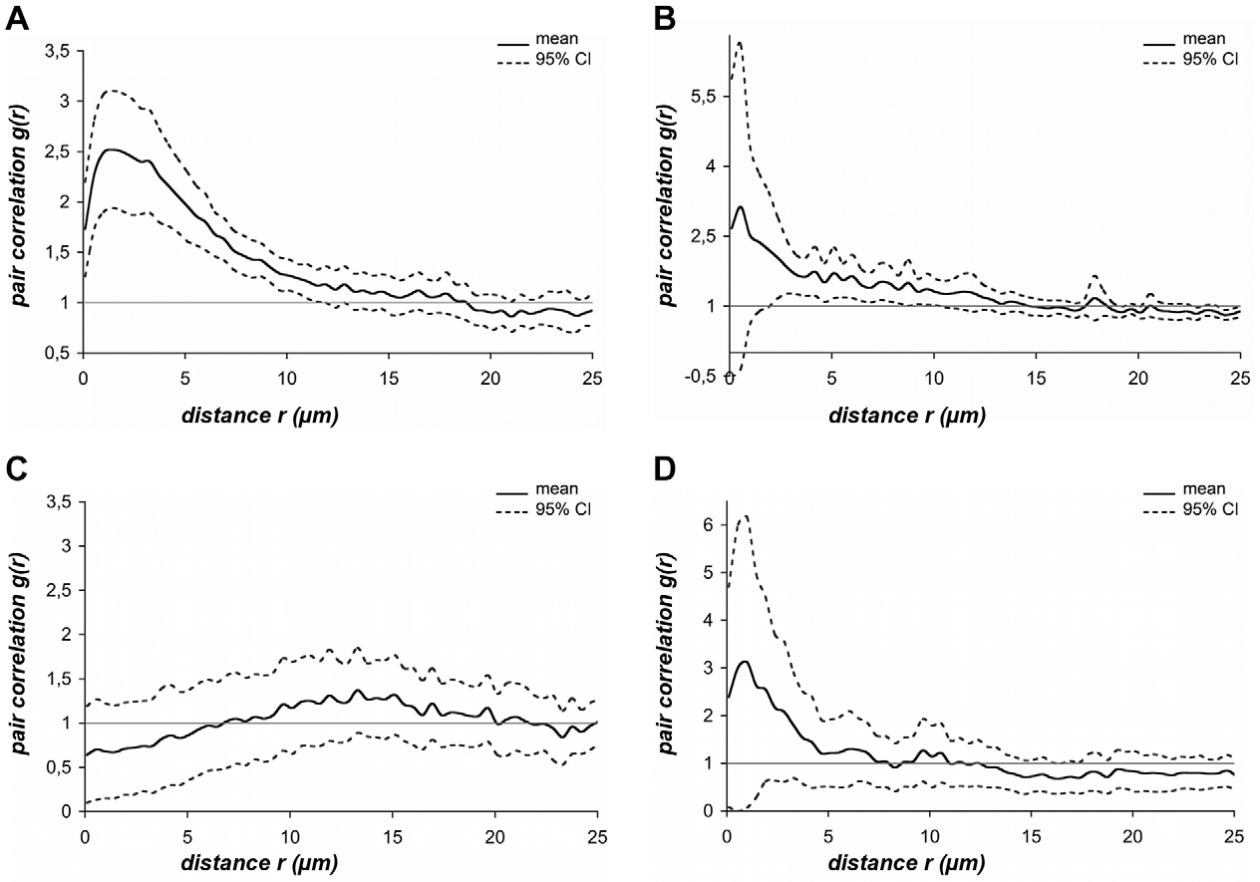
Pair cross correlation results. The mean PCC function *g*(*r)* (continuous line) and the 95% confidence interval (dotted lines) are plotted against distances *r* spaced at intervals of ∼0.5 µm. The dashed horizontal reference line on the level of *g*(*r*) = 1 corresponds to the value of randomness and provides an internal ‘null hypothesis’ for testing attraction or repulsion between cellular units. (A) Representative, individual-related PCC of *T. forsythia* and *F. nucleatum/periodonticum* calculated for 25 images obtained from patient 01. A pronounced peak of 2.5 PCC values at 1.5 µm indicated co-localization of *T. forsythia* and *F. nucleatum/periodonticum* cells within short distances from 0–6 µm. (B) Outlier evaluation. PCC of *T. forsythia* and *F. nucleatum/periodonticum* calculated for 25 images obtained from patient 10. An initially prominent peak was in contrast to Figure 3A embedded in a wide CI, which lower boundary (−95%) dropped below the reference line, indicating a high variance in PCC values within the first 3 µm. (C) Representative, individual-related PCC of *P. gingivalis* and *P. intermedia* calculated for 32 images obtained from patient 05. *P. gingivalis* and *P. intermedia* cells are randomly distributed within the entire distance range. (D) Outlier evaluation. PCC of *P. gingivalis* and *P. intermedia* was calculated for 30 images obtained from patient 04. Similar to the outlier results of *T. forsythia* and *F. nucleatum/periodonticum* (Figure 3 B), the high variance of PCC values at distances <4.7 µm allowed no valid analysis for patient 04. Above 4.7 µm the curve of *P. gingivalis* and *P. intermedia* oscillated around the reference line surrounded by a relatively narrow CI, whose lower limit remained below the PCC value g(r) = 1. These characteristics indicated random spatial distribution at distances >4.7 µm in contrast to the curve shown in Figure 3 B, whose PCC values decreased constantly.

Separate sets of random images were taken for each of the eight patients positive for *T. forsythia* and *F. nucleatum/periodonticum* for spatial analysis of these bacteria. From a total of 476 recorded micrographs, 199 were used for determining PCC values. Seven of the eight PCC curves for *T. forsythia* and *F. nucleatum/periodonticum* clearly showed co-localization of these species. The PCC plot of patient 01 (Figure 3A), based on a set of 25 randomly taken images, is described in detail as a representative example. Since in our samples *F. nucleatum/periodonticum* cells as the largest morphotype reached up to 10 µm in length, the spatial arrangement analysis of *daime* was set to plot the PCC function *g(r)* against distances *r* between 0 and 25 µm. The PCC curve showed a pronounced peak and a tight 95% CI. Following the mean PCC values, the lower CI (−95%) remained above the reference line *g*≡1 from 0 to 11 µm. Thus, two important criteria for co-localization were met within short distances between the two populations: (i) A pronounced peak of the mean PCC values along with (ii) a narrow CI whose lower boundary was clearly greater than 1.

These findings indicated tight spatial clustering of *T. forsythia* and *F. nucleatum/periodonticum* within a distance range from 0–6 µm. Similar PCC values were obtained for six of the other patients (data not shown). For only one patient (Figure 3B), however, a higher variance of the PCC values at short distances was observed and thus, for this specific sample co-aggregation of the two populations could not be confirmed unambiguously.

### Analysis of *P. gingivalis* and *P. intermedia*

Hybridization with probes POGI/PRIN resulted in strong hybridization signals suitable for quantitative analysis for six different subjects (18 subgingival plaque carriers). In contrast to the micrographs obtained for *T. forsythia* and *F. nucleatum/periodonticum*, the qualitative visual assessment of images showing *P. gingivalis* and *P. intermedia* was ambivalent due to a high variability of the observed distribution patterns. Within the same FOV seemingly repulsive and attractive localization patterns were found in close proximity. As exemplified by an image obtained from patient 03 (Figure 2A), the distribution of both species suggested a nearly constant distance between their microcolonies, which would indicate a repulsive spatial arrangement. Within a distinct region of the biomass, however, *P. gingivalis* and *P. intermedia* were located in direct vicinity, overgrowing each other (inset in Figure 2A). Furthermore, occurrence of both species in the same FOV was less common than for *T. forsythia* and *F. nucleatum/periodonticum*. Thus, mere visual observation was insufficient to characterize the spatial distribution of *P. gingivalis* and *P. intermedia*, and statistical spatial analysis was required.

### Individual-related spatial analysis of *P. gingivalis* and *P. intermedia*

In total 175 images obtained from six different patients were used for spatial arrangement analysis of *P. gingivalis* and *P. intermedia*. The PCC curve for patient 05, whose image set consisted of 32 micrographs (Figure 3C), is representative for most of the image sets analyzed. The mean PCC fluctuated slightly above and below the reference line [*g*(*r*) = 1], intersecting this line several times. As the CI limits consistently enclosed the reference line, this curve clearly indicated random spatial distribution of the two bacterial species.

The PCC curves obtained for four of the six patients were consistent with random distribution within the analyzed distance range of 0–25 µm. For patients 01 and 04, however, the curves were different within the first 4 µm. The PCC curve calculated for patient 04 reached a pronounced peak at a distance of 1 µm with 95% CI spanning a broad range above and also below the reference line (Figure 3D). The PCC curve obtained for patient 01 was similar (data not shown). Due to the high variance of the PCC values for patient 01 and 04 within the first 4 µm, these curves do not unequivocally indicate co-aggregation of the two populations despite the peak of the mean PCC. In contrast, the CI limits suggest a random distribution also in these two cases. To assess the effect of these outliers on the patient group as a whole, all individual-related PCC curves for *P. gingivalis* and *P. intermedia* were merged by statistical evaluations.

### PCC analysis of consolidated patient groups

**Figure 4:**
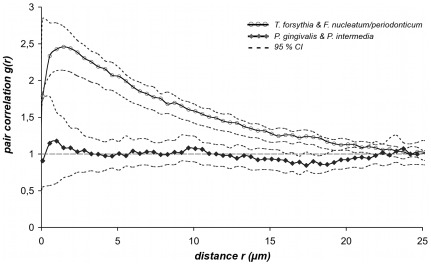
PCC curves of consolidated patient groups for *T. forsythia* versus *F. nucleatum/periodonticum* (*n* = 8) and *P. gingivalis versus P. intermedia* (*n* = 6). To compare the results of co-localization and randomness the patient group of each bacterial pair was merged by calculating the arithmetic mean curve with respective 95% CI by applying the equation 95% CI = m±1.96* SEM. The mean PCC curve for *T. forsythia* versus *F. nucleatum/periodonticum* (gray line, unfilled circles) and *P. gingivalis*/versus *P. intermedia* (black line, diamonds) were plotted with their respective 95% CI (dotted lines) against distances *r* in a range from 0–25 µm. The two curves were clearly distinguished from each other, by peak-levels and by convergence with the reference line. The fact that the lower CI of the co-localized bacteria was evidently separated from the upper CI of the randomly distributed pair of species within a wide range of 0–19 µm indicated a significant difference of both curves.

For a statistical comparison of the results obtained for the *T. forsythia versus F. nucleatum/periodonticum* and *P. gingivalis versus P. intermedia* pairs, we calculated for both group means the respective 95% CI as described in Methods. Both consolidated PCC curves for *T. forsythia* and *F. nucleatum/periodonticum* (*n* = 8) and *P. gingivalis* and *P. intermedia* (*n* = 6) were plotted with their respective 95% CI against a distance range of 0–25 µm (Figure 4). The two curves were significantly different in terms of peak heights and progression relative to the reference line. The curve for *T. forsythia* and *F. nucleatum/periodonticum* exhibited a strong and statistically significant peak within very short distances (peak maximum at 1.46 µm), clearly suggesting co-localization of these two populations. In contrast, the curve for *P. gingivalis* and *P. intermedia* confirmed random distribution by fluctuating around the reference line without any significant peak. The clear separation of the lower CI of the co-localized bacterial species from the upper CI of the randomly distributed organisms within 0–19 µm shows that the two curves are significantly different and that the two population pairs follow different spatial arrangement patterns in the biofilm.

## Discussion

Bacterial interactions play important roles in the pathogenic potential of polymicrobial medical biofilms such as the subgingival biofilms in periodontal disease. Several decades of periodontal research were characterized by different approaches to reveal the interaction patterns within the microbial community associated with disease. While providing valuable information these previous studies were intrinsically limited by: **i)** Focusing on pairwise *in vitro* interactions of planktonic cells, **ii)** employing biofilm models with cultivable species, **iii)** examining the *in vivo* distribution of subgingival species on a qualitative level only or **iv)** using disrupted rather than intact biofilm samples. Our present study adds a new level of understanding to these earlier studies by analyzing *in vivo* grown subgingival biofilms of a statistically significant number of patient samples with a novel method to quantify the nature of colonization patterns. This approach enables for the first time the rigorous statistical verification of microbial interactions, in subgingival plaque, that were previously proposed from *in vitro* experiments or from qualitative (intrinsically subjective) microscopic biofilm observations.

The image analysis software *daime* was extended by a new feature of its spatial arrangement tool, which was required for the correct image analysis of sectioned oral biofilms. The added functionality (reference space mask images) is not specific for medical biofilms but extends to all cases where biomass does not cover the whole area of the images to be analyzed. This includes also environmental biofilm samples.

By addressing the aforementioned methodical issues, we successfully analyzed the spatial arrangement patterns of *T. forsythia*/*F. nucleatum/periodonticum* and *P. gingivalis*/*P. intermedia* within *in vivo* grown specimen obtained from 10 GAP patients. These four oral bacterial species play important roles in a medical context and have been implicated as putative periodontal pathogens.

### *T. forsythia and F. nucleatum/periodonticum*

*T. forsythia* and *F. nucleatum/periodonticum* were chosen as one of the test pairs in this study, since they have been proposed to adhere to each other and form synergistic relationships [26], [54]. In addition, these two oral species are members of the red and orange complexes, respectively, which indicate disease correlation. According to extensive co-occurrence studies by Socransky and coworkers [17], members of the red complex (such as *T. forsythia*) are strongly correlated with pocket depth and severity of disease, while the orange complex species (such as *F. nucleatum*) precede the red complex and were proposed to facilitate colonization of red complex bacteria. The initial visual assessment (Figure 1) resembled recent findings by Zijnge *et al.* [33] who observed frequent close association of *T. forsythia* and *F. nucleatum*. These authors also observed these species to reside predominantly in the “intermediate layer” of the examined tooth attached biofilms. These qualitative impressions of co-localization of *T. forsythia* and *F. nucleatum* and previous *in vitro* studies suggesting interaction of these species [26], [33], [54], [56] were confirmed by the quantitative evaluation carried out in this study. Altogether, the results of past research and the data reported here strongly suggest a positive biological interaction between these two important disease-related oral bacteria.

### *P. gingivalis* and *P. intermedia*

Similar to *T. forsythia* and *F. nucleatum/periodonticum*, our second test pair *P. gingivalis* and *P. intermedia* are also classified as members of the red and orange complexes, respectively. In contrast to *T. forsythia* and *F. nucleatum/periodonticum*, however, reports about a possible mutualistic relationship of these organisms have been controversial. Based on *in vitro* co-aggregation experiments of *P. gingivalis* vesicles with *P. intermedia* cells, Kamaguchi *et al.* [42] concluded that *P. gingivalis* and *P. intermedia* physically interact via a HPG17 domain protein. In contrast, Kolenbrander and coworkers [23], [43] did not observe such interaction between these two species. Consistently, visual inspection of *in vivo* grown subgingival biofilm sections did also not indicate co-aggregation, because *P. gingivalis* and *P. intermedia* appeared to grow predominantly in distinct microcolonies ([33] and this study, Figure 2). The quantitative spatial analysis (Figures 3 and 4) has resolved the controversy about *P. gingivalis* and *P. intermedia* by confirming random distribution of these bacteria, relative to each other, in the *in vivo* grown biofilm. Thus, at least the spatial arrangement of these two species does not point at any specific biological interaction (mutualism or repulsion) between them. We assume that the co-existence of *P. gingivalis* and *P. intermedia* in the same parts of the subgingival biofilm is caused by other and not yet identified factors.

### Possibilities and Limitations

In this study, we show that digital image analysis can be used to objectively quantify and describe medical biofilm architecture. These *daime*-based analyses of bacterial distribution and interaction patterns in biofilms could be combined with the recently developed CLASI-FISH approach [40] to enable simultaneous investigation of the relationships between multiple bacterial species. This would allow for a comprehensive investigation of oral biofilms. However, one has to state, that this analysis depends on the choice of probes and their specificity and is therefore not an ‘open end’ approach. Furthermore, the sampling strategy using the carrier system allows for analysis of in vivo grown subgingival biofilms, that might be different from biofilms that have been developed in the periodontal pocket over weeks and months. On the other hand this carrier system enables for standardized sampling that can be repeated in the same patient to analyze oral biofilm development over time. A useful tool to analyze this would be the digital stratification of the biofilm with defined distance to the surface to allocate bacterial species in distinct layers of the biofilm.

### Conclusion

This “proof of principle” study performed the first quantitative analysis of bacterial spatial arrangement patterns within *in vivo* grown medical biofilms and clearly distinguished co-localization from random spatial distribution of different populations. The results are consistent between patients, and thus demonstrate the highly organized architecture of subgingival biofilms. Importantly, the methods used in this study are exclusively culture-independent. Hence, they can be applied for validating *in vitro* experiments by analyses of naturally grown biofilms as well as for the *de novo* investigation of yet uncultured microorganisms. Understanding the interactions among oral bacteria is an important prerequisite for the development of targeted therapeutic concepts. The quantitative characterization of spatial localization patterns has the potential to reveal previously overlooked interactions, whose nature can subsequently be studied by using culture-independent methods that analyze microbial physiology on the single-cell level [57]. This approach is not limited to the analysis of subgingival biofilms, but can efficiently be applied to other medical or environmental samples.
